# Editorial: Current trends in muscle diseases and their treatment strategies

**DOI:** 10.3389/fcell.2025.1660074

**Published:** 2025-07-23

**Authors:** Zihang Guo, Shoulong Deng

**Affiliations:** Institute of Laboratory Animal Sciences, Chinese Academy of Medical Sciences and Peking Union Medical College, Beijing, China

**Keywords:** neuromuscular disorders, muscle diseases, diagnostic techniques, personalized medicine, pig models

Muscle diseases encompass a broad spectrum of disorders, including genetic, degenerative and age-associated neuromuscular conditions, all characterized by muscle degeneration, functional impairment and in many cases, premature mortality. These diseases impose significant clinical burdens, with cardiorespiratory failure, fatal motor neuron loss and frailty in aging populations. Advanced diagnostic strategies, ranging from genetic testing and biomarker analysis to advanced imaging techniques have improved early and accurate detection. Meanwhile, therapeutic breakthroughs such as gene therapy and epigenetic modulators are transforming patient care. However, therapeutic development critically depends on translational animal models, particularly porcine models replicating human pathology. This Research Topic tries to present a comprehensive overview of the latest progress in muscle diseases.

Duchenne muscular dystrophy (DMD) is a fatal neuromuscular disease caused by loss-of-function mutations in the X-linked dystrophin gene and characterized by progressive muscle degeneration, loss of ambulation and premature death from cardiac or respiratory failure. Remarkable advancements have been made in both diagnostic methods and targeted therapies for DMD. Current diagnostic approaches combine genetic testing for exon deletion/duplication analysis with ancillary methods which include biomarker such as serum creatine kinase, muscle biopsy for dystrophin analysis and histopathology, advanced imaging techniques (muscle MRI and emerging MSOT), and cardiac monitoring through echocardiography and ECG. These ensure accurate diagnosis and comprehensive disease characterization. Recent breakthroughs in DMD treatment include exon-skipping therapies, gene editing-mediated reading frame restoration, and artificial chromosome transfer. AAV-based micro-dystrophin delivery (Elevidys) partially restores dystrophin expression and slows down disease progression, which is FDA-approved for ambulatory children aged ≥6. Antisense oligonucleotides (e.g., eteplirsen, viltolarsen, golodirsen, casimersen) promote exon skipping and produce functional truncated-dystrophin, which benefit patients with specific mutations.

Emerging research has expanded to epigenetic modifications. Histone deacetylases (HDACs) have been shown to be hyperactive in patients with DMD and contribute to the pathology, HDAC inhibition has arisen as a potential therapeutic option. The HDAC inhibitor givinostat is the first nonsteroidal treatment approved by FDA for DMD patients ≥6 years old. Aartsma-Rus published a study investigating the multi-targeted mode of givinostat in treating DMD. Givinostat has demonstrated the potential to addresses the pathophysiological cascade of DMD by targeting key pathological events originated by the lack of dystrophin. Preclinical and clinical trials confirmed givinostat works by inhibiting HDACs activity to promote histone acetylation and improve chromatin structure, while significantly slows disease progression by reducing fat infiltration, inflammation and fibrosis, promoting muscle regeneration and improving muscle function with a favorable safety profile. Furthermore, improved anesthesia protocols address perioperative risks. Lian et al. provide a systematic review of the pharmacological interventions for anesthesia and sedation in patients with DMD. They confirmed that patients with DMD are more sensitive to neuromuscular blocking agents (NMBAs), leading to delayed onset time, prolonged recovery time from anesthesia and risks such as malignant hyperpyrexia with volatile anesthetics. Precautions for DMD patients should include quantitative neuromuscular, electrocardiographic monitoring and rapid airway protection throughout anesthesia. Regional anesthesia was deemed relatively safer compared with general anesthesia. This review emphasized avoiding succinylcholine to prevent known anesthetic hazards such as rhabdomyolysis or hypercalcemia and highlighted the efficacy of Dantrolene for reversing malignant hyperpyrexial response to anesthesia.

Porcine DMD models, which replicate the biochemical, clinical, and pathological features of human patients with accelerated disease progression and early cardiac involvement, have been instrumental in evaluating novel diagnostic tools like multispectral optoacoustic tomography (MSOT) for non-invasive disease monitoring and testing therapeutic strategies. Despite challenges like high cost, porcine model of DMD remains invaluable for optimizing therapies and diagnostics and developing personalized care strategies.

Amyotrophic lateral sclerosis (ALS) is an adult-onset neurodegenerative disorder characterized by progressive degeneration of upper and lower motor neurons, leading to muscle weakness and atrophy, dysphagia or respiratory muscle palsy and remains incurable with an average survival of 2–5 years post-diagnosis. Current drugs like riluzole and edaravone offer only modest benefits. ALS is a multifactorial disease, involving oxidative stress, mitochondrial dysfunction, protein misfolding and metabolic disturbances. More than 30 ALS-related genes, including superoxide dismutase 1 (SOD1) gene, have been reported. Diagnosis of ALS relies on clinical assessments and biomarkers, such as elevated TDP-43 levels in peripheral blood mononuclear cells (PBMCs). Therapeutic strategies under investigation include metabolic interventions to restore glucose utilization in skeletal muscle. Fenili et al. provide a review and explore the potential of physical exercise as a co-adjuvant therapy for ALS. While intense exercise may pose risks, moderate and tailored exercise such as swimming have shown neuroprotective effects in animal models by improving glucose metabolism, mitochondrial function, and antioxidant defense. Resistance and endurance training may enhance quality of life and slow down functional decline in human. This review underscores the need for personalized exercise protocols in ALS. Transgenic pig models such as hSOD1G93A and mSOD1-Tg pigs replicate human ALS pathology, including biomarker dynamics and metabolic dysfunction. These models have emerged as transformative tools in testing diagnostics and therapies for ALS to enhance translational relevance.

Sarcopenia, an age-related syndrome characterized by progressive loss of muscle mass, strength, and function, has emerged as a critical health concern. Two representative surveys leveraging China Health and Retirement Longitudinal study (CHARLS) database revealed risk factors and predictive models in vulnerable groups and offered insights for early intervention. Yang et al. proved that sarcopenia mainly affects middle-aged and older Chinese women, closely related to age, waist, education, marriage, area, stroke, physical pain, depression, and region. They offer an effective tool to help clinicians better screen potential female patients. Qiao et al. developed a predictive model for sarcopenia risk in middle-aged and older adults with diabetes mellitus (DM) and validated a strong accuracy. Eight key predictors were identified: age, residence, BMI, diastolic blood pressure, cognitive function, activities of daily living (ADL), peak expiratory flow (PEF), and hemoglobin. This model serves as a practical tool for early identification of high-risk individuals and facilitates timely interventions to mitigate sarcopenia progression in diabetic populations. Zhong et al. employed bioinformatics and systems biology approaches to explore the shared pathogenic mechanisms between COVID-19 and sarcopenia. They identified 66 common differentially expressed genes (DEGs), including 15 hub genes. Enrichment analysis revealed functions and pathways between two diseases. Key regulators like FOXC1 and hsa-mir-155-5p were identified, and immune infiltration analysis highlighted the correlation between hub genes and immune factors. They also proposed potential therapeutic drugs and showed that valinomycin PC3 UP is the best candidate for the treatment of sarcopenia and COVID-19. Finally, they demonstrated ALDH1L2 and KLF5 showed the best diagnostic potential for COVID-19 and sarcopenia. These findings suggest shared pathways in COVID-19 and sarcopenia, offering insights for early diagnosis, effective treatment and targeted therapies.

Beyond above muscle diseases, several other neuromuscular disorders present unique pathological mechanisms and therapeutic challenges. Fibrosarcoma, an aggressive and poorly understood soft tissue sarcoma, poses significant therapeutic challenges. Radzka et al. evaluated the potential role of natural NF-κB inhibitors in fibrosarcoma. The results showed selective cytotoxicity toward cancer cells by inducing apoptosis and indicated that CAPE, biochanin A, and CurE could inhibit actin polymerization and disrupt the cytoskeleton of cancer cells. Cellular stress and vacuolation as well as metabolic changes are more pronounced in cancer cells compared to normal cells. This research supports the effect and safety of these natural compounds with low-toxicity as anticancer agents, particularly in the treatment of fibrosarcoma. Lu et al. provide a review and explore the role of mitochondrial defects in sporadic inclusion body myositis (sIBM), a subtype of idiopathic inflammatory myopathies (IIM) with unique pathological features such as muscle inflammation, rimmed vacuoles, and protein aggregation within the myofibers. Key mitochondrial abnormalities including large-scale mitochondrial DNA deletion, aberrant protein aggregation, and slowed organelle turnover are more prominent in sIBM than in other types of IIM, indicating that non-immune tissue dysfunction might contribute to the disease’s onset as patients with sIBM are refractory to conventional immunosuppressant treatment. They also discuss potential mitochondrial-targeted therapies, such as antioxidants and mitophagy inducers like Urolithin A. Understanding mitochondrial dysfunction in sIBM could pave the way for novel treatments targeting organelle health. Spinal muscular atrophy (SMA) is an autosomal recessive genetic disorder marked by progressive, symmetrical muscle weakness and atrophy. Jieda et al. provide a case report and describe a child with initial limb hypotonia and abnormal signal changes in brain MRI. Genetic testing ultimately confirmed the diagnosis of SMA. This report highlights the rarity of brain MRI abnormalities in SMA and the importance of early intervention, which may aid early diagnosis alongside genetic testing. Pathogenic variations in gene encoding the skeletal muscle ryanodine receptor (RyR1) are associated with malignant hyperthermia (MH) and RYR1-related myopathies (RYR1-RM). R615C porcine model, the first established preclinical model, has been instrumental in understanding pathomechanisms and testing potential therapeutics like dantrolene.

In addition to conventional treatment methods, recent studies demonstrate the successful generation of humanized skeletal muscle in MYF5/MYOD/MYF6-null pig embryos using blastocyst complementation with human induced pluripotent stem cells (hiPSCs). These findings offer potential of producing exogenic tissues for xenotransplantation to address challenges in muscle diseases treatment and highlight the feasibility of interspecies chimeras in regenerative medicine.

In summary, the landscape of muscle disease research is rapidly evolving driven by innovations in diagnostics, targeted therapies, and preclinical models. These advances underscore the importance of multidisciplinary collaboration. While challenges such as therapeutic accessibility and complexity of disease heterogeneity remain, future efforts should focus on personalized interventions, translational research and animal models to bridge the gap between bench and bedside, ultimately offering hope for improved outcomes and enhancing the quality of life for patients worldwide.

